# Peripheral perfusion index percentiles for healthy newborns by gestational age and sex in China

**DOI:** 10.1038/s41598-020-60741-9

**Published:** 2020-03-06

**Authors:** Xiao-jing Hu, Jin-xiang Ding, Ying Wang, Conway Niu, Yi Zhang, Qu-ming Zhao, Wei-li Yan, Yun Cao, Guo-ying Huang

**Affiliations:** 1 Children’s Hospital of Fudan University, Shanghai, China; 2 Pujiang Health Care Center, Shanghai, China; 3 Shanghai Songjiang District Maternal and Child Health Hospital, Shanghai, China; 4 Aberdeen Maternity Hospital, Aberdeen, Scotland; 5 Shanghai Key Laboratory of Birth Defects, Shanghai, China

**Keywords:** Paediatric research, Hypoxia

## Abstract

Peripheral perfusion index (PPI) percentiles for newborns serve as an important observation tool in clinical practice, but research pertaining to reference ranges are lacking. The aim of this study was to establish PPI percentiles for healthy newborns by gestational age and sex at 24–48 hours of life. We conducted an observational study and examined PPI values at 24–48 hours of life in 3814 asymptomatic newborns born between 35 and 41 weeks gestation who did not need medical treatment from June 1, 2016 to May 31, 2017 at two maternity hospitals in Shanghai. Linear regression analysis was carried out on the associations between PPI values and variables such as gestational age, sex, and birthweight. Pre-ductal PPI values linearly increased with gestational age (β: 0.072; 95% CI: 0.037, 0.107; P = 0.000). Post-ductal PPI values were also mainly related to gestational age (β: 0.051; 95% CI: 0.018, 0.085; P = 0.003). Smoothed reference curves for pre- and post-ductal PPI values by gestational age and sex were derived from LMS Chart Maker. Our study is the first study to establish PPI percentiles curves for healthy newborns by gestational age and sex at 24–48 hours of life. Further research is required for the implementation of PPI curves into clinical practice.

## Introduction

Peripheral perfusion index (PPI) is a reliable indicator to evaluate pulse intensity from the photoelectric plethysmographic signal of a pulse oximeter^1^. PPI reflects the ratio of the pulsatile (AC) to non-pulsatile (DC) signal components of blood flow in the peripheral tissue^2^. PPI thus non-invasively provides a continuous and real-time perfusion status of the selected monitoring site^1,3^.

In both preterm and term infants, a reduced PPI, when considered alongside other parameters such as oxygen saturation and pulse rate, suggests deterioration of newborn health^4^. In subclinical choroamnionitis, PPI can accurately identify affected newborns and influence immediate outcomes^5^. In a Swedish PPI study of healthy full-term newborns, PPI has also been suggested as a screening tool for critical left heart obstruction^6^. PPI for predicting low superior vena cava flow in very low birthweight infants may be very useful in detecting cardiovascular changes^7^. As such, PPI is increasingly considered as a simple and non-invasive objective measurement to improve the detection sensitivity of important neonatal complications^8^.

However, to effectively use PPI as a marker of neonatal health its reference range must be established. The PPI is affected by many factors. The median PPI of hemodynamically stable preterm infants was determined through a study by Cresi *et al*. assessing 30 newborns. PPI was registered at different times after birth and it was concluded that the median PPI was 0.9% on day 1 of life, 1.22% on day 3 of life, and 1.35% on day 7 of life. The authors suggested that postnatal age should be considered when using PPI for clinical evaluation^9^. A study by Vidal *et al*. also established different PPI values for preterm infants, but in those with persistent ductus arteriosus (PDA) of hemodynamic importance. They found that PPI was mainly affected by the age of the newborn, not by the flow pattern of the PDA. The median PPI at day 1 was 0.7% (0.5–1.05%) and 1.5% (1.0–2.0%) at day 7 (P < 0.01)^10^. It has been confirmed that PPI values can be affected by pulse oximeter sensor positioning, especially in very preterm infants, in whom the PPI of the upper limbs (right upper limb = 0.92% and lower limb = 0.69%, mean difference = 0.24%, *P* < 0.001) is higher, which may be explained by the changes in circulation *in vivo*^11^. It was also found that there were more infants with PDAs in this group.

To date, there have been no reports on the normal range of PPI values from China. PPI values may be related to the age and temperature of the newborn and the position of pulse oximeter sensor. This study addresses this gap in data through measurement of PPI at two maternal hospitals in Shanghai. Participants were born between 35–41 weeks’ gestation and had their PPI measured at 24–48 hours of life. PPI values obtained were used to establish PPI reference curves based on gestational age and sex, which can provide a reference for obstetricians and neonatologists to monitor the health status of newborns.

## Material and Methods

### Study design and participants

This was an observational study which aimed to monitor physiological parameters at 24–48 hours of life in asymptomatic newborns born at 35–41 weeks’ gestation via uncomplicated vaginal delivery or elective caesarean section who did not need medical support. All infants in the study were consecutively born in the newborn nursery at two public maternity hospitals with approximately 4000 annual deliveries between June 1 2016 and May 31 2017 who underwent standard postnatal care. Immediately after birth, infants were placed under a radiant warmer and transferred to the postnatal ward after the infant was considered stable. The inclusion criteria for this study are liveborn and healthy individuals born between 35 and 41 weeks’ gestation. We excluded stillbirths, infants who died within 2 days after delivery, infants with unstable vital signs who were admitted to the neonatal intensive care unit, and those who could not stay with his/her mother, as well as those who were not able to be routinely discharged home.

### Ethical approval and informed consent

The methods were carried out in accordance with approved guidelines and regulations. Ethical approval to conduct the research was obtained from the Research Ethics Committee of the Children’s Hospital of Fudan University, Shanghai, China. Informed consent was obtained from the parent(s) of all participants.

### Data collection

On the day after delivery, a resident pediatrician, nurse, and researcher were present and physiological parameters of the neonate, including oxygen saturation (SpO_2_), PPI, and temperature were recorded at 24–48 hours of life in the postnatal ward. Both pre- and post-ductal SpO_2_ were monitored by using two motion-tolerant pulse oximeter monitors (Rad 7, Masimo Corporation, Irvine, California, USA) simultaneously. Reusable probes with disposable wraps were placed on the right palm or wrist for pre-ductal and on either foot for post-ductal saturation monitoring. When the infants were lying quietly in the supine position, oximetry monitoring with stable tracings of the SpO_2_ and PPI were obtained. The SpO_2_ and PPI values were recorded when both pre- and post-ductal pulse oximetry showed stable waveforms and the correlating PPI values. The values were monitored repeatedly three times and the data were averaged for each infant. Limits of PPI values identified to be invalid which required repeated measurement were ≤0.02 and ≥20 and simultaneous absence of SpO_2_ values^12^. In addition, sex, gestational age, time of measurement, temperature of measurement, delivery mode, birthweight, and maternal health status were also recorded.

### Statistical analysis

Continuous variables are described as the median and interquartile range while categorical variables are shown as numbers and percentages. Characteristic data were compared by the Mann-Whitney U test for continuous variables and the χ^2^ test or Fisher’s exact test for categorical variables. Multiple linear regression was performed to investigate the association between PPI values and gestational age by adjusting sex, mode of delivery, birthweight, temperature, and SpO_2_. Statistical analyses were performed using SPSS (Version 22.0; IBM Corp., Chicago, Illinois, USA). Statistical significance was defined as *P* < 0.05, 2-tailed.

We estimated and presented in percentiles the distribution of PPI values for each 1-week increment in gestational age from 35 to 41 weeks based on sex. Furthermore, we plotted the curves along the 3rd, 10th, 25th, 50th, 75th, 90th, and 97th percentiles using Cole’s Lambda Mu Sigma (LMS) method^13^. The LMS approach initially estimates the three parameters of Box-Cox transformation of the distribution of the measurement. The L determines a nonlinear transformation of PPI, such that its distribution approximates the normal distribution. The M stands for the mean of that normal distribution, and S for the coefficient of variation. The three parameters are constrained to change smoothly as the covariate changes^14^. L, M, and S correspond to the following formulas: Z = (X/M)L − 1/LS, where X indicates the measured value of PPI and the centile = M* (1 + L* S* Zα)1/L, where Zα is the z-score that corresponds to a given percentile (e.g. 3rd, 10th, 25th, 50th, 75th, 90th, or 97th). The Z-score is a measure of the distance in SDs of a sample from the mean^15^. To create smoothed percentile curves for the 3rd, 10th, 25th, 50th, 75th, 90th, and 97th percentiles, LMS Chart Maker light version 2.54 (Medical Research Council, London, UK) was used.

## Results

**Figure 1:**
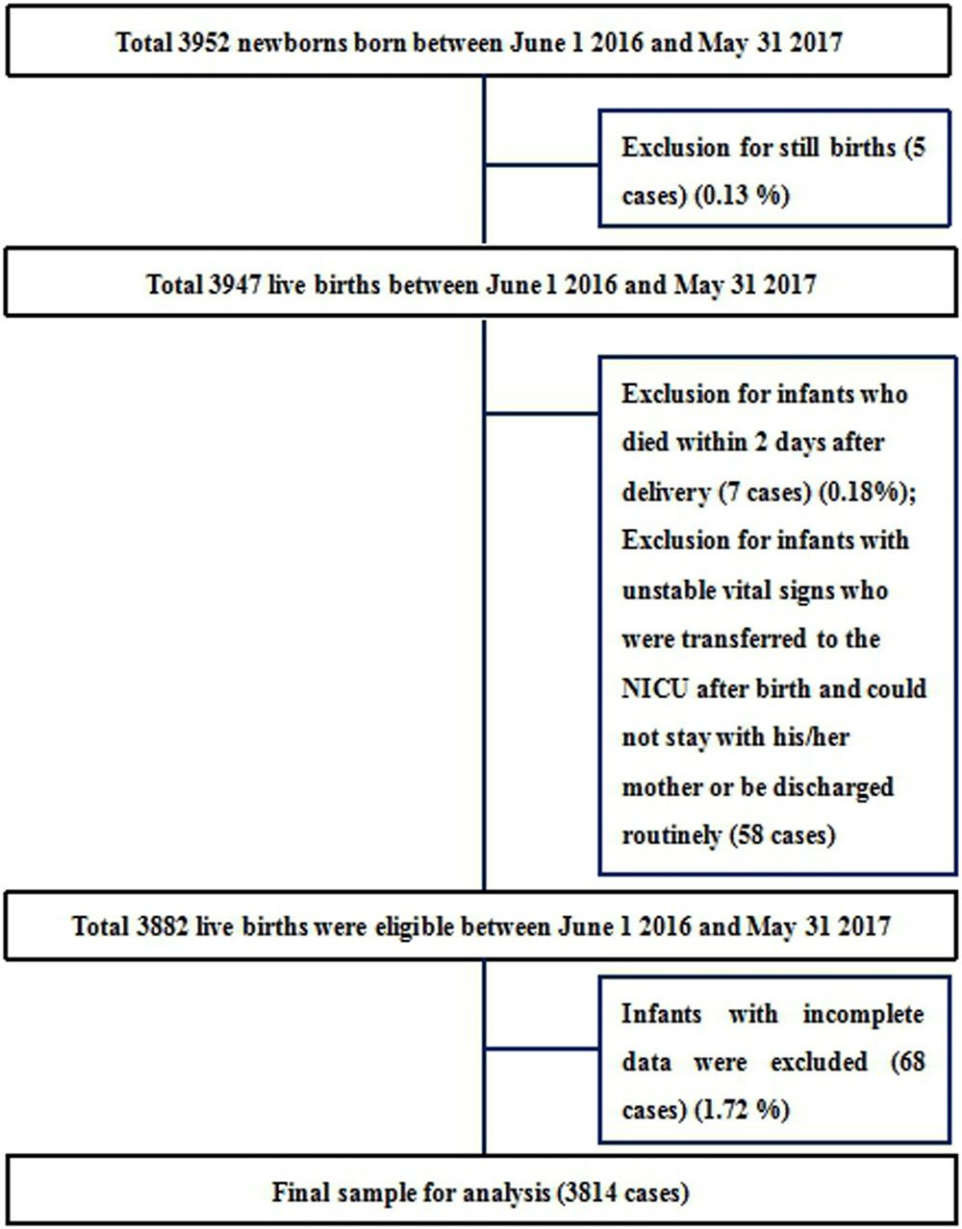
Exclusion flow of the participants.

In total, 3952 newborn babies of 35–41 weeks’ gestation were delivered at the study sites, of whom 3814 were eligible and recruited. Our exclusion process is outlined in Fig. 1. Median age at measurement was 37.00 hours of life (IQR: 28.75 h–44.00 h). Median pre-ductal PPI value was 2.10 (IQR: 1.60–3.30) and post-ductal PPI value was 2.30 (IQR: 1.70–3.40) (Z=−5.24, *P* < 0.05). The mean gestational age was (39.07 ± 1.21) weeks. The mean pre- and post-ductal PPI values and gestational age were calculated according to sex, method of delivery, measurement time, SpO_2_ values, and temperature as presented in Table 1. When pre-ductal SpO_2_ was less than 97%, mean post-ductal PPI was higher (*P* < 0.05). However, when post-ductal SpO_2_ was less than 97%, mean pre-ductal PPI was higher (*P* < 0.05).

**Table 1:** PPI values and gestational age of newborns according to variables.

Variables	No. of cases (%)	Mean of pre-ductal PPI (%) (s.d.)	Mean of post-ductal PPI (%) (s.d.)	Gestational age (weeks) (s.d.)
Total of eligible births	3814 (100.00)	2.54 (1.33)	2.63 (1.28)	39.07 (1.21)
**Sex**				
Male	2056 (53.91)	2.52 (1.33)	2.61 (1.28)	39.01 (1.21)**
Female	1758 (46.09)	2.57 (1.34)	2.65 (1.28)	39.13 (1.21)**
**Mode of delivery**				
Caesarean section	1127 (29.55)	2.59 (1.41)	2.66 (1.29)	38.99 (1.24)**
Virginal	2687 (70.45)	2.53 (1.31)	2.62 (1.27)	39.10 (1.20)**
**Hypothermia** (**<36.5 **°**C)**				
Yes	919 (24.10%)	2.57 (1.37)	2.62 (1.30)	39.08 (1.23)
No	2895 (75.90%)	2.54 (1.33)	2.64 (1.27)	39.06 (1.21)
**Pre-ductal SpO**_**2**_**<97%**				
Yes	1391 (36.47%)	2.53 (1.29)	2.71 (1.29)**	39.02 (1.26)
No	2423 (63.53%)	2.55 (1.36)	2.59 (1.27)**	39.09 (1.18)
**Post-ductal SpO**_**2**_**<97%**				
Yes	959 (25.14%)	2.70 (1.35)**	2.60 (1.30)	39.05 (1.27)
No	2855 (74.86%)	2.49 (1.33)**	2.64 (1.27)	39.07 (1.19)
**Measure time** (**24–36 h)**				
Yes	1768 (46.36%)	2.55 (1.34)	2.62 (1.29)	39.07 (1.18)
No	2046 (53.64%)	2.54 (1.34)	2.64 (1.27)	39.07 (1.24)

**Means *P* < 0.01.

We undertook univariate and multivariate analyses of pre-ductal PPI values and from Tables 2 and 3 it can be seen that pre-ductal PPI values were not associated with sex (β: 0.051; 95% CI: −0.035, 0.136; *P* = 0.247), measurement time (β: 0.000; 95% CI: −0.005, 0.006; *P* = 0.862), temperature at measurement (β: −0.046; 95% CI: −0.181,0.088; *P* = 0.498), delivery mode (β: 0.070; 95% CI: −0.024,0.163; *P* = 0.143), birthweight (β: −0.000; 95% CI: 0.000,0.000; *P* = 0.651), or post-ductal SpO_2_ (β: 0.000; 95% CI: −0.002, 0.002; *P* = 0.997). However, pre-ductal PPI increased linearly with gestational age (β: 0.072; 95% CI: 0.037, 0.107; *P* = 0.000). Post-ductal PPI values increased linearly with gestational age (β: 0.051; 95% CI: 0.018, 0.085; *P* = 0.003) and pre-ductal SpO_2_ (β: −0.029; 95% CI: −0.048, −0.010; *P* = 0.003).

**Table 2:** Univariate and multivariate analysis of Pre-ductal PPI values in healthy newborns.

Items	Univariate analysis			Multivariate analysis		
	β	95%CI	*P*	β	95%CI	*P*
Gender	0.060	−0.025, 0.145	0.170	0.051	−0.035, 0.136	0.247
Gestational age	0.070	0.035, 0.105	0.000^**^	0.072	0.037, 0.107	0.000^**^
Pre-ductal SpO_2_	−0.018	−0.038, 0.002	0.074	−0.020	−0.040, 0.001	0.056
Post-ductal SpO_2_	0.000	−0.002, 0.002	0.965	0.000	−0.002, 0.002	0.997
Measurement time	0.001	−0.005, 0.006	0.772	0.000	−0.005, 0.006	0.862
Temperature at measurement	−0.049	−0.183, 0.085	0.471	−0.046	−0.181, 0.088	0.498
Delivery mode	0.060	−0.033, 0.153	0.205	0.070	−0.024, 0.163	0.143
Birth weight	−0.000	0.000, 0.000	0.802	−0.000	0.000, 0.000	0.651

*Means *P* < 0.05; **means *P* < 0.01.

**Table 3:** Univariate and multivariate analysis of Post-ductal PPI values in healthy newborns.

Items	Univariate analysis			Multivariate analysis		
	β	95%CI	*P*	β	95%CI	*P*
Gender	0.039	−0.042, 0.121	0.342	0.035	−0.047, 0.117	0.400
Gestational age	0.050	0.017, 0.084	0.003^**^	0.051	0.018, 0.085	0.003^**^
Pre-ductal SpO2	−0.028	−0.047, −0.009	0.004^**^	−0.029	−0.048, −0.010	0.003^**^
Post-ductal SpO2	0.000	−0.002, 0.002	0.684	0.000	−0.002, 0.002	0.701
Measurement time	0.002	−0.003, 0.007	0.507	0.002	−0.004, 0.007	0.554
Temperature at measurement	−0.024	−0.153, 0.104	0.710	−0.023	−0.151, 0.106	0.729
Delivery mode	0.036	−0.053, 0.125	0.429	0.040	−0.049, 0.130	0.374
Birth weight	0.000	0.000, 0.000	0.435	0.000	0.000, 0.000	0.533

**Means *P* < 0.01.

**Figure 2:**
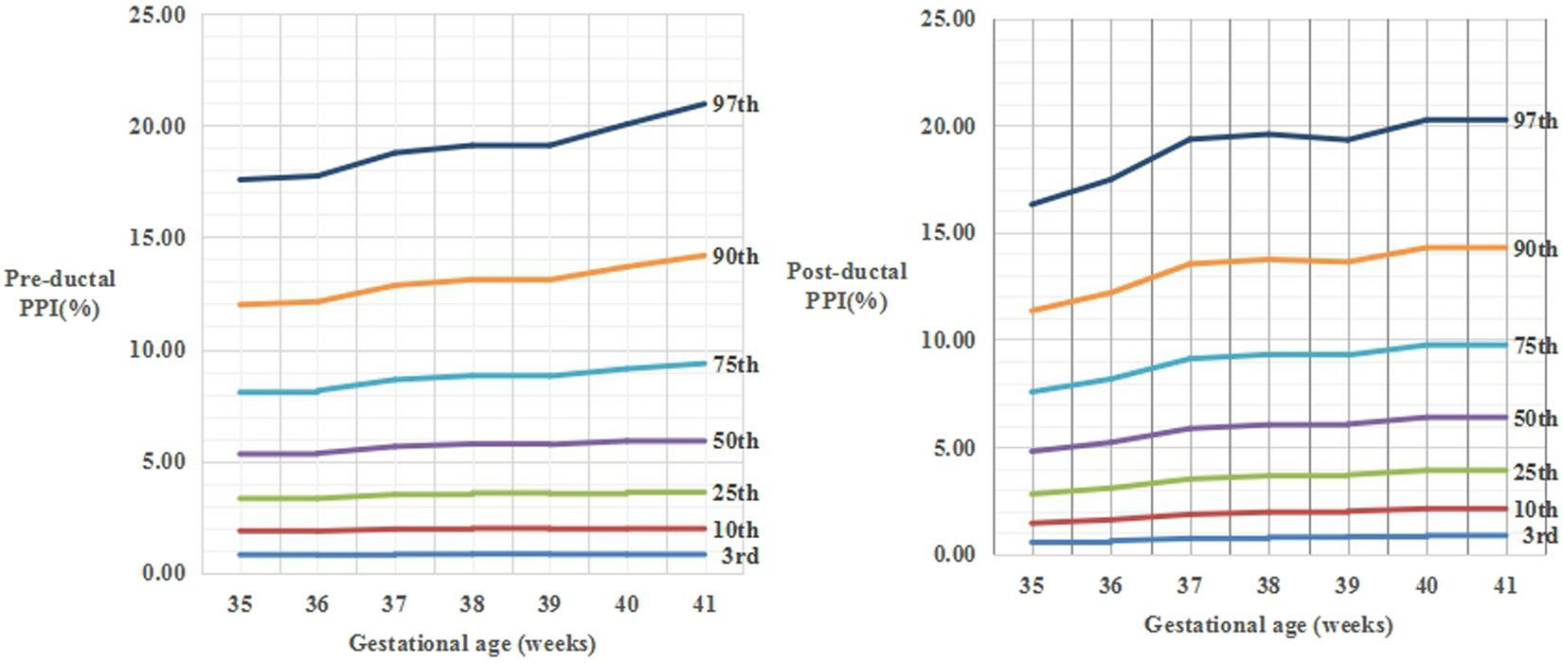
The percentile curves of pre-ductal PPI values (A) and post-ductal PPI values (B) by gestational age for all newborns.

**Figure 3:**
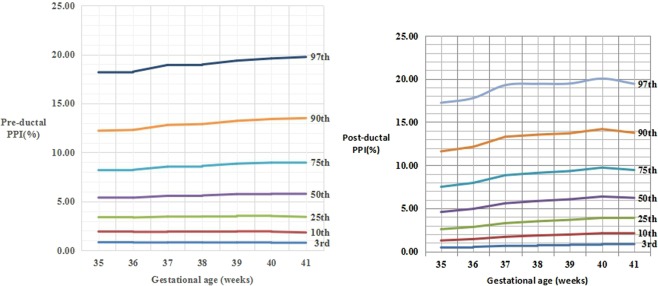
The percentile curves of pre-ductal PPI values (A) and post-ductal PPI values (B) by gestational age for male newborns.

**Figure 4:**
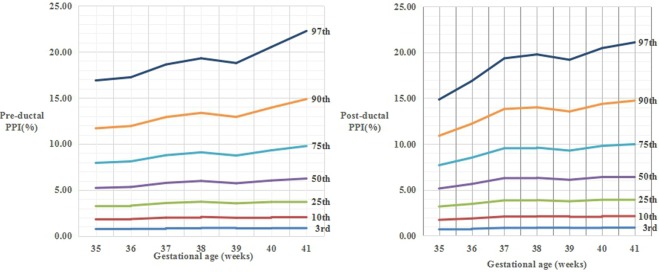
The percentile curves of pre-ductal PPI values (A) and post-ductal PPI values (B) by gestational age for female newborns.

Smoothed reference curves for pre- and post-ductal PPI values by gestational age and sex at the 3rd, 10th, 25th, 50th, 75th, 90th, and 97th percentiles are shown in Figs. 2–4. The LMS parameters for the pre- and post-ductal PPI values at the 3rd, 10th, 25th, 50th, 75th, 90th, and 97th pre- and post-PPI percentiles by gestational age and sex are presented in Tables 4 to 9, respectively.

**Table 4:** The pre-ductal PPI curves L, M and S parameters and percentile pre-ductal PPI by gestational age in all newborns.

Age	n	L	M	S	Percentiles						
					3rd	10th	25th	50th	75th	90th	97th
35	40	−0.13	1.99	0.48	0.80	1.07	1.45	1.99	2.76	3.90	5.59
36	41	−0.09	2.02	0.49	0.79	1.07	1.46	2.02	2.81	3.96	5.63
37	241	−0.06	2.15	0.49	0.82	1.13	1.55	2.15	2.99	4.20	5.93
38	855	−0.04	2.19	0.49	0.83	1.15	1.58	2.19	3.06	4.28	6.01
39	1219	−0.02	2.20	0.50	0.82	1.14	1.58	2.20	3.06	4.29	6.02
40	962	0.01	2.30	0.51	0.81	1.15	1.63	2.30	3.23	4.55	6.38
41	456	0.05	2.38	0.54	0.79	1.15	1.66	2.38	3.39	4.81	6.78

GA, gestational age (e.g. 36 weeks represent 36 weeks + 0 day to + 6 days). LMS, Lambda Mu Sigma.

**Table 5:** The post-ductal PPI curves L, M and S parameters and percentile post-ductal PPI by gestational age in all newborns.

Age	n	L	M	S	Percentiles						
					3rd	10th	25th	50th	75th	90th	97th
35	40	0.31	1.99	0.53	0.55	0.89	1.37	1.99	2.78	3.76	4.97
36	41	0.29	2.13	0.52	0.62	0.98	1.48	2.13	2.96	4.01	5.29
37	241	0.25	2.36	0.51	0.73	1.12	1.65	2.36	3.26	4.40	5.82
38	855	0.21	2.38	0.49	0.79	1.17	1.69	2.38	3.28	4.42	5.85
39	1219	0.20	2.36	0.48	0.81	1.19	1.70	2.36	3.23	4.32	5.70
40	962	0.19	2.48	0.48	0.86	1.26	1.78	2.48	3.38	4.52	5.97
41	456	0.16	2.46	0.48	0.86	1.25	1.77	2.46	3.37	4.53	6.02

GA, gestational age (e.g. 36 weeks represent 36 weeks + 0 day to + 6 days). LMS, Lambda Mu Sigma.

**Table 6:** The pre-ductal PPI curves L, M and S parameters and percentile pre-ductal PPI by gestational age in male newborns.

Age	n	L	M	S	Percentiles						
					3rd	10th	25th	50th	75th	90th	97th
35	22	−0.22	2.00	0.49	0.83	1.09	1.46	2.00	2.80	4.02	5.97
36	20	−0.16	2.03	0.50	0.81	1.08	1.47	2.03	2.84	4.07	5.95
37	144	−0.10	2.12	0.50	0.81	1.11	1.53	2.12	2.98	4.25	6.13
38	496	−0.06	2.15	0.50	0.81	1.11	1.54	2.15	3.02	4.27	6.08
39	648	−0.02	2.22	0.51	0.81	1.13	1.58	2.22	3.11	4.37	6.15
40	502	0.05	2.26	0.52	0.78	1.12	1.60	2.26	3.18	4.45	6.19
41	224	0.12	2.30	0.53	0.73	1.09	1.60	2.30	3.25	4.53	6.24

GA, gestational age (e.g. 36 weeks represent 36 weeks + 0 day to + 6 days). LMS, Lambda Mu Sigma.

**Table 7:** The post-ductal PPI curves L, M and S parameters and percentile post-ductal PPI by gestational age in male newborns.

Age	n	L	M	S	Percentiles						
					3rd	10th	25th	50th	75th	90th	97th
35	22	0.27	2.00	0.60	0.47	0.81	1.31	2.00	2.92	4.11	5.62
36	20	0.27	2.10	0.57	0.54	0.90	1.41	2.10	3.01	4.18	5.65
37	144	0.25	2.30	0.54	0.66	1.05	1.58	2.30	3.25	4.46	5.98
38	496	0.23	2.35	0.51	0.74	1.12	1.65	2.35	3.26	4.43	5.89
39	648	0.23	2.39	0.49	0.79	1.18	1.70	2.39	3.27	4.38	5.76
40	502	0.22	2.47	0.47	0.86	1.26	1.78	2.47	3.35	4.46	5.84
41	224	0.16	2.38	0.47	0.87	1.24	1.73	2.38	3.23	4.31	5.68

GA, gestational age (e.g. 36 weeks represent 36 weeks + 0 day to + 6 days). LMS, Lambda Mu Sigma.

**Table 8:** The pre-ductal PPI curves L, M and S parameters and percentile pre-ductal PPI by gestational age in female newborns.

Age	n	L	M	S	Percentiles						
					3rd	10th	25th	50th	75th	90th	97th
35	18	−0.02	1.97	0.48	0.76	1.05	1.44	1.97	2.72	3.76	5.19
36	21	−0.01	2.02	0.48	0.77	1.07	1.47	2.02	2.78	3.84	5.30
37	97	−0.01	2.18	0.48	0.84	1.15	1.59	2.18	3.00	4.14	5.71
38	359	−0.02	2.26	0.48	0.88	1.20	1.64	2.26	3.11	4.28	5.92
39	571	−0.04	2.17	0.49	0.83	1.14	1.57	2.17	3.01	4.19	5.85
40	460	−0.04	2.33	0.51	0.85	1.19	1.66	2.33	3.28	4.63	6.58
41	232	−0.04	2.46	0.54	0.85	1.21	1.72	2.46	3.53	5.09	7.39

GA, gestational age (e.g. 36 weeks represent 36 weeks + 0 day to + 6 days). LMS, Lambda Mu Sigma.

**Table 9:** The post-ductal PPI curves L, M and S parameters and percentile post-ductal PPI by gestational age in female newborns.

Age	n	L	M	S	Percentiles						
					3rd	10th	25th	50th	75th	90th	97th
35	18	0.47	1.96	0.41	0.69	1.03	1.46	1.96	2.55	3.21	3.96
36	21	0.37	2.17	0.44	0.75	1.12	1.59	2.17	2.87	3.70	4.66
37	97	0.27	2.43	0.46	0.84	1.24	1.76	2.43	3.26	4.28	5.53
38	359	0.19	2.43	0.47	0.86	1.25	1.76	2.43	3.30	4.40	5.77
39	571	0.16	2.34	0.47	0.84	1.21	1.70	2.34	3.19	4.27	5.64
40	460	0.16	2.48	0.48	0.87	1.26	1.78	2.48	3.40	4.58	6.09
41	232	0.17	2.54	0.50	0.86	1.26	1.81	2.54	3.51	4.76	6.36

GA, gestational age (e.g. 36 weeks represent 36 weeks + 0 day to + 6 days). LMS, Lambda Mu Sigma.

## Discussion

Pulse oximeter waveforms contain additional information that have not been fully exploited despite its great clinical potential. Most studies have been focused on the establishment of PPI parameters for early detection of critical conditions in patients^6,8,10,16–18^. However, few studies have discussed the distribution of PPI in healthy term and late preterm newborns between 24–48 hours after birth. This paper is the first to present sex-specific pre- and post-PPI percentiles of infants born between 35 and 41 weeks’ gestation at 24–48 hours of life. These percentile charts can help clinicians and researchers identify high risk neonates earlier. However, these percentile charts only can be used for newborns with gestational age between 35 and 41 weeks at 24–48 hours after birth.

In the current study, we set very strict inclusion criteria such as gestational age between 35 and 41 weeks, measurement time between 24–48 hours of life, with measurements being repeated thrice, and calculating the average value as result for each measurement, and ensuring that infants were kept in a quiet environment in the supine position. Because PPI values have been shown to vary greatly during the transition period immediately after birth^19^ and that it is routine for newborns to be discharged from the maternity hospital before 48 hours of age, the observation period of 24–48 hours after birth was chosen. Strict exclusion criteria were adhered to, such as gestational age of <35 or ≥42 weeks or those who required admission to the NICU. These strict criteria were adhered to in hopes of obtaining accurate results for this specific population during a specified time of life. The mode of delivery has previously been reported to have no relationship to PPI^20,21^, and the current study validates this finding.

Granelli *et al*.^16^ found a median PPI of 1.68 in the right hand (IQR: 1.18–2.46) and 1.71 in any foot (IQR: 1.20–2.50) for newborns within 1 to 120 hours of birth. Our results were slightly higher, which may be explained by the greater range in the time of measurement in their study as compared to ours, with measurements being taken as early as 1 hour of age. Furthermore, in our study, we obtained a significantly higher post-ductal PPI, which was different from those reported by Hakan *et al*.^22^, which reported a median pre-ductal PPI as 1.31 (IQR: 0.97–1.81) and a significantly lower median post-ductal PPI of 0.88 (IQR: 0.59–1.32). However, they reported the mean pre-ductal SpO_2_ to be 94% (92–96%), lower than the mean post-ductal SpO_2_ of 96% (94–98%). In our study, the median pre-ductal SpO_2_ was 97% (96–99%), which was also significantly lower than post-ductal values of 98% (96–99%). The median SpO_2_ and the median PPI values in our study were higher than those reported by Hakan *et al*. Post-ductal PPI was higher than pre-ductal PPI in our study, which shows that PPI distribution may be relatively consistent in asymptomatic newborns between 24 and 48 h of life. These results may be due to differences in the characteristics of the participants; the infants in that study were born earlier than the infants in our study.

Tables 2 and 3 show that pre-ductal PPI values increased linearly with gestational age (β: 0.072; 95% CI: 0.037, 0.107; *P* = 0.000). Post-ductal PPI values linearly increased with gestation at birth (β: 0.051; 95% CI: 0.018, 0.085; *P* = 0.003). This suggests that PPI is related to gestational age. An interesting finding was that those with lower pre-ductal SpO_2_ were found to have greater post-ductal PPI values (β: −0.029; 95% CI: −0.048, −0.010; *P* = 0.003). A possible explanation for this is that in our study, the baby’s hands were exposed, while the feet were wrapped in blankets. Hence, although the body temperature was normal, the temperature of the hands would have been presumably cooler than the feet. Because pulsatility decreases with vasoconstriction and increases with vasodilatation, changes in PPI reflect changes of peripheral vascular tension^23^. In this scenario, when the hands were cooler, the pulsatile component of PI decreased, whereas the nonpulsatile component remained unchanged, which may have led to the lower PI value^1^. PPI is mainly affected by the blood flow at the monitoring site rather than the level of arterial oxygen saturation, which reflects the real-time fluctuation in peripheral blood flow^24^. PPI is different from SpO_2_ in that in the absence of pressure, neonatal peripheral perfusion is higher than oxygen supply in the skin, so when the clinical condition of the infant deteriorates, cardiac output is redistributed to vital organs such as the heart, brain, and adrenal glands^25^. Therefore, PPI monitoring plays an important role in evaluating the overall clinical status of newborns.

Many studies focus on using PPI to predict life-threatening diseases. For example, for very low birth weight infants, when PPI value is less than 0.44, it may indicate that blood flow in the superior vena cava is less than 40 ml/kg/min^7^. Granelli *et al*.^16^ suggested using a PPI value of 0.7 as a method to evaluate whether newborns had left heart obstructive lesions, which was also a supplementary way to screen for congenital heart disease. However, it is necessary to know the normal range for infants of specific gestational age at specific postnatal periods. Since PPI is related to gestational age, we used LMS software to plot the PPI curve based on gestational age. In order to differentiate between male and female infants accurately, sex-based PPI curves were drawn for clinical reference. The next anticipated step is to use the reference curve to predict neonatal clinical status.

During the study period, no healthy infants were identified to have significant neonatal complications before discharge. Most newborns born in the hospital had continuing observation and regular care until discharge. We also carried out sufficient follow up for these infants to confirm whether any had other medical issues after discharge. No infants were found to have been later diagnosed with any disease in the follow-up clinic at 42 days of life.

There are some limitations to this study. The study population was primarily within Shanghai. Further multi-center studies in different regions are needed to investigate economic, geographical, and other factors of the population. A future study to verify the sensitivity of the present PPI reference charts in detection of unwell term newborns may be appropriate. We collected data on consecutive births, so fewer babies with smaller gestational weeks (35 weeks) and larger gestational weeks (42 weeks) were included only to observe trends.

In conclusion, for the first time, our study plots percentile curves of PPI by gestational age and sex for newborns. Newborn-specific PPI curves will be important for obstetricians and pediatricians to identify high-risk neonates. Further research on the application of PPI curves in clinical practice is needed to verify its effectiveness.
